# 2007 The clinical research operations program: Educating clinical research staff

**DOI:** 10.1017/cts.2018.228

**Published:** 2018-11-21

**Authors:** Peg Tsao, Veronica Haight, Ashley Dunn, Lisa Jackson, Steven Goodman

**Affiliations:** Stanford University School of Medicine

## Abstract

OBJECTIVES/SPECIFIC AIMS: The Clinical Research Operations Program is a free educational program designed to educate clinical research personnel on the conduct of clinical research (CR). The participant completes 16 required core sessions (24 h), 4 elective sessions (4 h), and passes the final exam to receive a certification in CR operations at Stanford. Sessions focus on the 9 domains of CR (established by the Joint Task Force for Clinical Trial Competency), such as Ethical & Participant Safety Considerations, Clinical Study Operations, & Data Management/Informatics. METHODS/STUDY POPULATION: Sessions are taught by volunteer lecturers. Participants may also attend the sessions without pursuing the certification. The program objective is to provide easy-access education in CR in order to increase regulatory compliance, staff retention, and improve CR at Stanford. The program targets CR coordinators, however, staff, postdocs, fellows, and faculty also participate. RESULTS/ANTICIPATED RESULTS: Since the program’s launch in January 2017, 119 individuals have enrolled in the certification program. The most represented group is the Department of Medicine. Sessions consistently reach their maximum with a waiting list. Each core session requires that the participant complete an evaluation (Likert scale, 1–5) of the registration process (4.5/5), the class environment (4.6/5), the presented content (4.5/5), and the instructor (4.6/5). Data from these evaluations are positive to date and is used to continually refine the program. DISCUSSION/SIGNIFICANCE OF IMPACT: N/A.

